# Seaweeds as Source of Bioactive Pigments with Neuroprotective and/or Anti-Neurodegenerative Activities: Astaxanthin and Fucoxanthin

**DOI:** 10.3390/md22070327

**Published:** 2024-07-22

**Authors:** Estela Guardado Yordi, Amaury Pérez Martínez, Matteo Radice, Laura Scalvenzi, Reinier Abreu-Naranjo, Eugenio Uriarte, Lourdes Santana, Maria Joao Matos

**Affiliations:** 1 Universidad Estatal Amazónica, 160101 Puyo, Ecuador; e.guardadoy@uea.edu.ec (E.G.Y.); amperez@uea.edu.ec (A.P.M.); mradice@uea.edu.ec (M.R.); lscalvenzi@uea.edu.ec (L.S.); rabreu@uea.edu.ec (R.A.-N.); 2 Departamento de Química Orgánica, Facultad de Farmacia, Universidade de Santiago de Compostela, 15782 Santiago de Compostela, Spain; eugenio.uriarte@usc.es (E.U.); lourdes.santana@usc.es (L.S.)

**Keywords:** seaweed, bioactive pigments, neuroprotection, neurodegenerative diseases

## Abstract

The marine kingdom is an important source of a huge variety of scaffolds inspiring the design of new drugs. The complex molecules found in the oceans present a great challenge to organic and medicinal chemists. However, the wide variety of biological activities they can display is worth the effort. In this article, we present an overview of different seaweeds as potential sources of bioactive pigments with activity against neurodegenerative diseases, especially due to their neuroprotective effects. Along with a broad introduction to seaweed as a source of bioactive pigments, this review is especially focused on astaxanthin and fucoxanthin as potential neuroprotective and/or anti-neurodegenerative agents. PubMed and SciFinder were used as the main sources to search and select the most relevant scientific articles within the field.

## 1. Introduction

In the last few decades, the incidence of neurodegenerative diseases worldwide has been very high, with increasing morbidity in Latin American and Caribbean countries [1]. Among them, dementia and its most common forms—Parkinson’s and Alzheimer’s diseases—constitute one of the main global causes of disability [2]. Therefore, it has been recognized as a public health priority by the *Organización Panamericana de la Salud* (OPS) and the World Health Organization (WHO) [3]. The growing morbidity and lack of efficient treatments have led to an increase in studies on the neuroprotective effects of natural bioactive compounds. The challenge is to find pharmacological or nutraceutical candidates that can be used in the treatment or prevention of these diseases. In this context, micro- and macroscopic algae are presented as a rich and varied source of bioactive compounds with biological activities [4,5]. The main advantages of these marine resources are their abundance and renewability, which are the reasons why they are being investigated as sustainable sources of safe bioactive compounds for pharmaceutical and nutraceutical applications [6,7,8].

Marine compounds, and especially those derived from algae, present improved characteristics in resistance to the adverse environment of deep oceans. Therefore, these molecules are more robust to adaptation, for example, to the absence of light and oxygen. Their diversity is really high due to the amount of different species available under the water. Being natural products, they are usually more potent and have fewer side effects compared to synthetic drugs [9,10].

Algae can be classified into two main groups: microalgae—which include blue–green algae, dinoflagellates, and Bacillariophyta (diatoms)—and macroalgae (seaweed), which include *Chlorophyceae* (green algae), *Phaeophyceae* (brown algae), and *Rhodophyceae* (red algae). *Rhodophyceae* are divided into three main groups according to the specific pigments that give them color [7,8]. In particular, microalgal phyla produce highly bioactive compounds with great chemical and pharmacological diversity. Red macroalgae are considered the most important source of biologically active metabolites compared to other classes of algae. Some of the biological activities attributed to them are the prevention of type II diabetes and anti-inflammatory, antioxidant (being related to preventive effects on cancer), antiproliferative, and antiangiogenic activities [6].

The research interest is based on the fact that, in 16 classes of algae, there are thousands of species of microalgae. Among them, three main groups stand out: *Chlorophyceae*, *Chrysophyceae*, and *Bacillariophyceae* (diatoms). The most studied for their biological implications are cyanobacteria, cyanophyceans, and diatoms. These algae have special characteristics: rapid growth, the capacity to form a layer on the surface of the water, and easy adaptation to adverse environmental conditions [11]. These are the reasons why cultivation to obtain high-value bioactive products is a fact today. For example, dehydrated algae biomass provides high performance and million-dollar profits in the main producing countries: Taiwan, China, Japan, and Germany, among others [12].

Although the diversity of biologically active compounds discovered in algae is wide and ranges from proteins to lipids, carotenoids, and polyphenols, among others, those considered pigments are the main focus of this review. Algae have been identified as natural producers of commercial bioactive pigments. Pigments provide color, making them widely used in industry, including the food and pharmaceutical industry, as additives or excipients, respectively. The bioactivity and extra benefit they can provide are a plus [13]. 

Natural marine biopigments are biosynthesized as part of the secondary and even primary metabolism [14]. There are three groups of pigments in seaweed: chlorophylls, carotenoids, and phycobiliproteins [15,16]. Some of the most studied are carotenoid-type pigments [6]. Among their multiple activities, antioxidant, anti-inflammatory, anti-carcinogenic, anti-angiogenic, anti-obesity, and neuroprotective properties stand out for their pharmacological interest [6]. These areas of research are currently being developed since these bioactive natural pigments could constitute candidates for the treatment or prevention of chronic diseases, through the development of either new drugs or nutraceuticals.

In the last five years, the number of investigations on carotenoids in marine substances has increased substantially, particularly on astaxanthin and fucoxanthin and their presence in algae. This article aims to offer a comprehensive and holistic approach to the analysis of the biopharmaceutical and nutraceutical potential of these two biopigments in the context of neurodegenerative diseases. In this way, it may be distinguished from previous research and reviews since it integrates the processes of cultivation, extraction, and applications as neuroprotective agents in a unified overview. An emphasis is placed on these biopigments not only as general bioactive compounds but specifically as derivatives of micro- and macroalgae due to their unique structural characteristics, thus enhancing their applicability in both the pharmaceutical and food industries. Furthermore, the incorporation of an updated review of ongoing human clinical trials, enriching their relevance for therapies against neurodegenerative diseases, has been included.

In summary, this review aims to present the current knowledge on pigments present in algae, specifically astaxanthin and fucoxanthin, with neuroprotective and/or anti-neurodegenerative bioactivity. The information obtained for this review was selected from scientific papers from recent years. PubMed and SciFinder were used to search and select the most relevant scientific articles within the field. To identify relevant clinical trials on the effects of fucoxanthin and astaxanthin in humans, we searched the EU Clinical Trials Register and ClinicalTrials.gov. Finally, other different sources were used to prepare this review, such as academic repositories from various universities and journal databases (ScienceDirect, Dialnet, Minerva, and Scielo, among others).

## 2. Pigments Present in Micro- and Macroscopic Algae

Among marine compounds, algae contain a wide variety of natural pigments that give them their characteristic colors and present different bioactivities. Natural pigments have multiple applications in different industries, including the pharmaceutical, food, and cosmetics industries. Currently, they are usually preferred over synthetic alternatives since they have a greater added value associated with their safety and biocompatibility [13]. The most studied are chlorophylls, phycobiliproteins, and carotenoids [2,16]. 

Carotenoids are a large family of highly studied chemical compounds. More than 600 pigments are known to be synthesized de novo in higher plants, fungi, bacteria, mosses, and algae. Their colors range from yellow to intense red. They are widely used as natural colorants. However, recently, the interest in carotenoids as possible health-promoting compounds has expanded considerably [17]. The chemical structure of this group of bioactive compounds is derived from lycopene. They are mostly hydrocarbons of 40 carbon atoms that contain two terminal ring systems linked via a polyene system [18]. 

Carotenes and xanthophylls are highlighted, amongst other carotenoids. Carotenes are composed only of carbon and hydrogen. Xanthophylls are oxygenated derivatives in which oxygen can be present in hydroxyl groups (zeaxanthin), as oxy groups (canthaxanthin), or in a combination of both (astaxanthin) (Table 1). It is known that each double bond of the carotene–polyene chain can exist as *cis* or *trans* geometric isomers, with the *trans* isomer being predominant in nature. The intense color of carotenoids is chemically explained by their long chains with conjugated double bonds. The absorption spectrum has maxima whose wavelengths depend on the number of conjugated double bonds. The presence of these conjugated double bonds in its structure seems to be related to its antioxidant capacity, allowing it to neutralize free radicals and other reactive oxygen species [19].

Astaxanthin, fucoxanthin, lutein, canthaxanthin, zeaxanthin, and β-cryptoxanthin are the best-known carotenoids (Table 1) [6]. The potential benefits of marine carotenoids have been studied, particularly for astaxanthin and fucoxanthin, which are the most relevant in marine sources (details in Section 2.1) [20].

The green color of *Chlorophyceae* or green algae is due to the presence of chlorophylls A and B (Table 1) in similar proportions to non-aquatic plants. The greenish brown color of *Phaeophyceae* is attributed to fucoxanthin, together with chlorophylls A and C. Phycobilins, such as phycoerythrin and phycocyanin, are responsible for the characteristic color of *Rhodophyta*. Chlorophylls are photosynthetic pigments with a porphyrin structure that are involved in photosynthesis. There are several reported chlorophylls: chlorophylls A, B, C, D, and E and bacteriochlorophylls A, B, C, D, and E. Chlorophylls A and B are present in photosynthetic tissue at a ratio of a–b (3:1). Chlorophyll C is present in brown algae, dinoflagellates, and diatoms, among other sources. Chlorophyll D is found in some red algae, especially those that live in low-light environments. Chlorophyll E is found in xanthophyta algae. Finally, bactochlorophylls A, B, C, D, and E are found in photosynthetic bacteria, including the families *Chromatiaceae* and *Rhodospirillaceae* [21].

Other biopigments present in algae are phycobiliproteins, which are fluorescent proteins of different colors. They are responsible for the characteristic coloration of algae, and they play a fundamental role in capturing light during photosynthesis. Phycobiliproteins are found in both cyanobacteria and red algae (Rhodophyta). Both groups have diverse biological activities, which makes their commercial production of interest to the food, biomedical, and cosmetic industries. Currently, several patents have been generated that focus on reducing the environmental impact on the natural resources of macroalgae and on issues related to increasing their productivity [22]. The most abundant phycobiliproteins are phycocyanin—present in cyanobacteria (blue–green algae) and some red algae (macroalgae)—and phycoerythrin, found mainly in some red algae (macroalgae) and cyanobacteria (blue–green algae). Chemically, these pigments are nitrogenous compounds (Table 1).

Phycoerythrin is a phycobilin pigment present in red algae, which is why it has the same color. It is a substance that absorbs light and acts in conjunction with chlorophyll. Phycocyanin is a blue phycobilin pigment, a conjugated chromoprotein that is present in blue–green algae. It is known for its ability to bind to pigment and capture light. The two main classes of this protein are cyanophycocyanin and recombinant phycocyanin, which can be derived from cyanobacteria or other organisms through genetic engineering techniques. It is characterized by its spectral property of λmax at 620 nm, and it is composed of two polypeptides, the α unit and the β unit (equimolar proportions). Cyanophycocynin is found in several different species, including red algae, dinoflagellates, and cryptophytes [23].

A study carried out in coastal areas of Sri Lanka investigated the chemical composition of 15 species of macroalgae, showing a higher protein content in brown algae, while the maximum contents of dietary fiber and ash were registered in the green ones. In that study, linoleic acid was reported as the dominant fatty acid of all macroalgae. The presence of minerals was higher in red macroalgae, but copper, zinc, and magnesium were in higher proportions in the green algae *Ulva lactuca* [24].

As mentioned, polyphenols have also been identified in marine macroalgae, having different structural characteristics and pharmacological potentials. Brown algae constitute the main source for its chemical extraction, and even those obtained from this raw material have higher antioxidant properties when compared to those obtained from red and green algae [25]. The flavonoids present in algae may contribute to their color in a similar way to that of land plants. It is proposed that some of the possible interactions responsible for the coloration are: (i) the interaction with other pigments, as the flavonoids present in algae can interact with other pigments, such as chlorophylls and carotenoids, thus modifying the color of the algae; (ii) chelating properties, as some flavonoids present in algae have the ability to bind to metals, which can influence the stability and color of the complexes formed; and (iii) antioxidant properties, as flavonoids can protect algae from damage caused via UV radiation and other environmental factors, influencing the production of protective pigments and, therefore, the color of the algae [26].

### 2.1. Astaxanthin and Fucoxanthin

**Figure 1:**
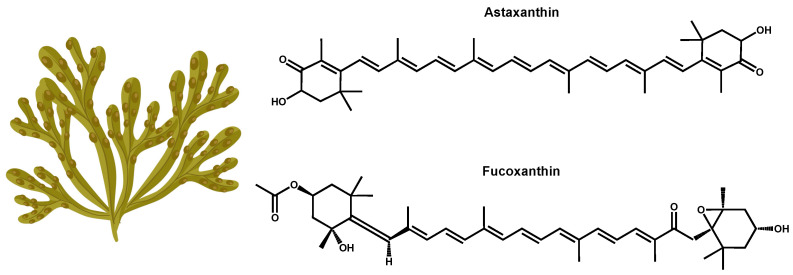
Chemical structures of astaxanthin and fucoxanthin and representation of their main natural origin. (Created by the authors on 20 July 2024, with the support of a free picture of a seaweed from Freepik).

Astaxanthin and fucoxanthin are at the top of the list of best-known carotenoids (Table 1 and Figure 1) [6]. Astaxanthin (C_40_H_52_O_4_, Table 1 and Figure 1) can be found in microalgae of the species *Haematococcus pluvialis* and *Chlorella zofingiensis*, both ideal strains for the commercial production of this compound [6,27]. *Haematococcus pluvialis* can accumulate concentrations of up to 5% of its weight on a dry basis, standing out as a model organism for the production of astaxanthin [27]. Other species of interest include *Neochloris wimmeri* and *Chlorococcum zofingiensis*, although their synthesis capacities are comparatively lower [28].

The in vitro synthesis of astaxanthin is possible, generating a racemic mixture of the two enantiomers and the *meso*, non-esterified form. Unlike those obtained through chemical synthesis, those derived from algae are always esterified [18]. Astaxanthin has a unique structure that is distinguished by having polar groups (hydroxyl and keto residues) at both ends of its polyene chain, which makes it structurally different from other carotenoids. This structural characteristic gives this pigment a notable antioxidant capacity and allows it to integrate into the cell membrane, facilitating several biological functions [29,30].

Fucoxanthin (C_42_H_58_O_6_, Table 1 and Figure 1) is a brown or light brown carotenoid pigment found in macroalgae and microalgae [31]. Fucoxanthin is the major carotenoid pigment in marine ecosystems, representing 10% of the total carotenoid production [32]. This pigment also belongs to the chemical family of xanthophylls, so it is an oxygenated derivative of carotenes, with distinctive structural characteristics to other carotenoids found in algae since it contains carbonyl, hydroxyl, carboxyl, and other chemical particularities, such as allene bonds, conjugated double bonds, and monoepoxy groups [33]. Due to the presence of these functional groups, this pigment may undergo oxidation and isomerization. This biopigment constitutes the main active ingredient of dietary algae [34]. When ingested, it is degraded via digestive enzymes that are secreted in the gastrointestinal tract to fucoxanthinol and amarouciaxanthin A [32]. The main species producing fucoxanthin are *Phaeodactylum tricornutum*, *Odontella aurita*, and *Chaetoceros calcitrans*, which stand out for their high content of this bioactive compound. 

In Table 2, values and classification of the important species of algae, according to the content of astaxanthin and fucoxanthin, can be observed.

### 2.2. Methods of Growing and Extracting Algae Pigments

Extraction and purification methods, an important part of pigments study, have been investigated in parallel to algae’s bioactivity [37]. The production of this type of high-quality bioactive molecule depends on an effective extraction and purification method, and its application is in correspondence not only with its possible bioactivity but also with its degree of purity. The purity of these biopigments is crucial, as impurities can reduce their efficacy. For example, in their commercial use as medicines or nutraceuticals, the elimination of non-GRAS solvents is a prerequisite [38].

**Figure 2:**
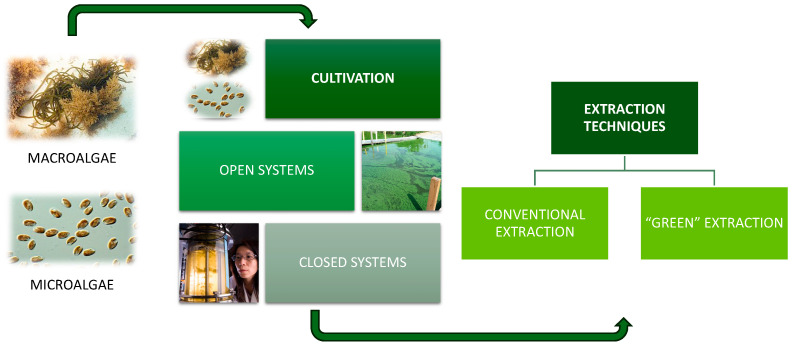
Methods of cultivation and extraction of algae pigments.

Algae cultivation has become an important area of research due to its potential as a sustainable source of pigments with therapeutic and nutritional applications. To obtain these biopigments, conventional extraction techniques continue to be used due to certain advantages such as low costs and operational simplicity (Figure 2). However, they also present several drawbacks, such as the use of large amounts of organic solvents, high extraction temperatures, and long times, as well as a low extraction yield [39]. To solve these problems, several “green extraction techniques” have been developed (Figure 2). These include microwave-assisted extraction, ultrasonic-assisted extraction, enzyme-assisted extraction, pressurized liquid extraction, and supercritical fluid extraction. These techniques may involve lower amounts of organic solvents, sustainable reagents, and much less energy. The main advantage is that they are environmentally friendly while improving extraction efficiency [39,40].

The choice of which algae species to cultivate depends directly on the final objective of the resulting biomass. For example, different species of algae are selected for the production of nutraceuticals and natural pigments based on their growth profiles, biochemical composition, and efficiency in producing the desired compounds. Factors such as the ability of algae to accumulate large amounts of specific bioactive compounds, their adaptability to different growing conditions, and their resistance to contaminants are also crucial in this selection [41]. In addition, the type of culture system must be considered. If it is open-air, it will depend on environmental and operational factors, as well as biological parameters [42]. On the other hand, if the system is indoor, monospecific crops isolated from the environment can be achieved [43].

The production of pigments via microbials is a promising technology that has been tested for production through microalgae. Microalgae cultivation traditionally occurs in outdoor systems. Specifically, open ponds have been used, for example, in the production of fucoxanthin from *P. tricornutum* since they allow mass production and have a low operational cost [44,45,46]. However, they involve the difficulty of limited control in facing environmental changes, the problem of mass transfer, and the loss of nutrients through evaporation [47]. On the other hand, the use of indoor systems provides more efficient and controlled production, such as the use of tubular photobioreactors, stirred tanks, and flat panels. Flat panels are desired to study and control factors such as light and the metabolic response of algae, in addition to allowing higher biomass production without the growth of contaminated aerobic microorganisms [48,49,50].

Microalgae are a rich source of carotenoids, as observed in various species. The carotene content in some genera of microalgae can reach up to 800 mg/g of dry weight, such as the case of *Euglena gracilis*, with 0.18 mg of xanthophylls/g in *Chlorella vulgaris* and up to 9% of the dry weight in *Dunaliella salina*. These yields favor industrial production [51]. Although many carotenoids are obtained synthetically due to low-cost production, legislative restrictions and consumer preferences have increased the use of natural sources. It is estimated that 100,000 tons of carotenoids are obtained annually from natural sources; their biotechnological production is being intensively investigated to improve the efficiency and sustainability of the process [52]. The determination of carotenoids in different matrices involves two steps. The first is sample preparation, extraction, and saponification, followed by separation, identification, and quantification. The factors to consider are light, since they are photosensitive, oxygen, and acids, which results in a certain degree of degradation and/or isomerization [17].

Recent updates to the production, extraction, and purification processes of microalgae pigments for commercial production indicate that they constitute a raw material for the production of value-added chemicals. To ensure the quality and purity of carotenoids, an accurate determination process is crucial. Anil et al. (2022) evaluated the extraction potential of organic solvents to obtain chlorophyll (A and B) and total carotenoids. A maximum astaxanthin yield of 0.82 μg/mL was obtained using dimethylsulfoxide for 20 min, which decreased by further increasing the extraction time. After the extraction process was supplemented with microwaves and ultrasound, the pigment yield increased [6].

Rich brown macroalgae such as *Laminaria japonica* and *Sargassum horneri* are also often used for fucoxanthin extraction [45,53]. The fucoxanthin concentration is higher in *Sargassum horneri*, at around 3700 mg/kg, exceeding by more than 2000 mg/kg the concentration found in common brown algae [54,55]. In countries such as China, Japan, and South Korea, fucoxanthin production has increased due to its high concentration in these algae [36]. Although fucoxanthin is mainly found in brown algae and diatoms, previous studies have shown that the fucoxanthin content of microalgae is higher than that of brown macroalgae [56]. The production of high-quality fucoxanthin depends on an effective extraction and purification method. These algae are grown in large open-air tanks or in closed photobioreactors, where parameters such as light, temperature, and nutrients are controlled to optimize their growth [37,57]. Various protocols have been used to recover and purify fucoxanthin from algae. These included centrifugal partition chromatography, column chromatography, microwave irradiation, pressurized liquid extraction, supercritical carbon dioxide extraction, ultrasound-assisted extraction, and traditional solvent extraction, followed by chromatographic methods. One efficient method is ultrasonic-assisted extraction, which uses ultrasound to break down cell walls and release bioactive compounds. Another advanced technique is supercritical CO_2_ extraction, which is more environmentally friendly and avoids the use of organic solvents [37].

As previously mentioned, astaxanthin is found in microalgae such as *Haematococcus pluvialis* and some other marine algae. The cultivation of *Haematococcus pluvialis* is commonly performed in closed photobioreactors to avoid contamination and control environmental conditions, such as light and CO_2_ concentration. In the growth phase, algae are grown under optimal nutrient and light conditions, and in the induction phase, they are stressed to increase astaxanthin production [58]. After the induction phase, the algae are harvested and dehydrated. Dehydration can be carried out by freeze-drying or spray-drying. The extraction of astaxanthin is carried out via solvent extraction, using ethanol, acetone, or hexane. As for fucoxanthin, ultrasound-assisted extraction is an efficient technique. Supercritical CO_2_ extraction is also used due to its ability to preserve the integrity of the compound and avoid the use of harmful solvents. Pigment production in algae is influenced by several biotic and abiotic factors. It happens naturally as part of its vegetative growth and is influenced by light, the length of the photoperiod, the availability of nutrients, temperature, pH, salinity, heavy metals, pesticides, etc. [6]. Although pigments’ main function is to help light collection during growth, they can also occur under stress conditions. It is known that astaxanthin is produced during high light and nitrogen stress, while salt stress triggers the synthesis of canthaxanthin [59].

Once grown, the seaweed is harvested and dried. Carotenoids can be easily degraded via exposure to light, high temperatures, or solvents, so the selection of the appropriate steps and procedures is crucial to maintaining their stability. The presence of water in the microbial biomass is considered unfavorable due to its hydrophobicity, which is why freeze-drying of the biomass is preferred, even though it prolongs the time and costs. Carotenoid samples should be protected from UV light [52]. Dehydration can be carried out by freeze-drying or air-drying, which facilitates the grinding of the dried material to a fine powder, the starting point for the extraction of fucoxanthin.

## 3. Use of Pigments as Nutraceuticals and Their Therapeutic Potential

As reported by Tavares et al., 2023, macroalgae can be considered functional foods, food supplements, or promising sources of bioactive compounds with beneficial effects on human health [60]. Pigments have acquired a fundamental role as commercial products. In particular, fucoxanthin has obtained authorization from the Food and Drug Administration (FDA) in the United States. Fucoxanthin seems to be able to control the glycemic index and also promote a reduction in cholesterol levels. Astaxanthin, fucoxanthin, and bixin have been mentioned as potential anti-amyloidogenic compounds, a key bioactivity for natural treatment of Alzheimer’s disease, which is the most prevalent neurodegenerative disorder in the elderly population. These molecules can inhibit the aggregation and assembly of β-amyloid, mitigating the neurodegenerative process [61]. The accumulation of toxic proteins and oxidative stress are key factors for neurodegenerative diseases. Carotenoids seem to be able to provide a protective effect due to their antioxidant properties and inhibition of neuroinflammation. Several sources of evidence have established the role of neuroinflammation in neurodegenerative diseases such as Alzheimer’s and Parkinson’s diseases. Inflammatory components such as microglia, astrocytes, the complement system, and cytokines are related to neuroinflammation in the central nervous system. In numerous studies, the levels of inflammatory cytokines are unusually high in samples of patients suffering from these pathologies [62].

### 3.1. Therapeutic Potential of Algae-Derived Pigments in Neurodegenerative Diseases

The therapeutic potential of pigments obtained from algae has aroused great interest due to their possible applications in the treatment and prevention of diseases, including anti-neurodegenerative/neuroprotective diseases. The combined effects of antioxidation and anti-inflammation are essential for neuroprotection, offering a promising approach to prevent or mitigate the impact of neurodegenerative diseases through the use of natural compounds derived from algae. Neutralizing free radicals reduces oxidative stress that can damage neurons and contribute to neurodegenerative diseases such as Alzheimer’s and Parkinson’s diseases [25]. By reducing oxidative damage, bioactive compounds help protect neuronal cells, maintaining their function and viability. Anti-inflammatory mechanisms promoting a reduction in inflammation, specifically a reduction in chronic inflammation in the brain, are a key factor in preventing the progression of neurodegenerative diseases. Likewise, the modulation of the immune system is beneficial since, by reducing the production of inflammatory mediators, neuronal tissue is, thus, protected.

There is evidence for the antioxidant and neuroprotective activity of the main pigments present in marine algae. Carotenoids exert medicinal and nutritional effects on the body mainly through dietary intake, and dietary nutrition is an important way to prevent and improve nervous system disorders [63]. The carotenoid astaxanthin is widely recognized for its potent antioxidant and anti-inflammatory properties [64]. It has higher antioxidant activity than a range of carotenoids, even higher than zeaxanthin or beta-carotene. Naturally occurring astaxanthin significantly reduces oxidative and free radical stress compared to synthetic astaxanthin [65]. It has the ability to cross the blood–brain barrier, allowing it to achieve the brain to reduce oxidative stress and neuronal inflammation [66,67,68]. In addition, it prevents lipid peroxidation in biological membranes, which is beneficial to human health [69]. Several studies have shown that astaxanthin can protect against different neurodegenerative conditions, including Alzheimer’s and Parkinson’s diseases, as well as other pathologies related to oxidative stress and inflammation. Its ability to activate the PI3K/Akt and MAPK/ERK pathways reinforces its neuroprotective role, increasing the expression of antioxidant enzymes and reducing lipid peroxidation and oxidative DNA damage [70].

Astaxanthin, biosynthesized via the microalgae *Haematococcus pluvialis*, presents an outstanding antioxidant activity [71]. The mechanisms of action that have been described are the neutralization of free radicals and other oxidants and the protection of the lipid bilayer from peroxidation. Furthermore, this pigment can neutralize superoxide radicals (O^2−^) and inhibit the activation of the transcription factor NF-κB, thereby reducing the production of proinflammatory cytokines. It also blocks cytokine production by modulating the expression of protein tyrosine phosphatase-1, which helps reduce inflammation and protect against oxidative stress [72,73,74,75]. It has been suggested that astaxanthin, due to its antioxidant properties, is, therefore, a neuroprotective agent [76]. In studies carried out on mice, astaxanthin proved to reduce the levels of oxidation markers in different regions of the brain, such as the cortex, striatum, hypothalamus, hippocampus, and cerebellum. Additionally, it increased the activity of antioxidant enzymes, such as SOD and CAT, and glutathione levels. This antioxidant effect was more pronounced in young mice, suggesting an age-dependent effect. Astaxanthin also showed neuroprotective effects in animal models of autism, reducing oxidative stress in the brain and improving altered behavior due to prenatal exposure to valproic acid [77,78]. Another study in mice, carried out by Lee et al. (2011), on the effects of astaxanthin using in vivo models of Parkinson’s disease suggested a potential neuroprotective effect. This study investigated how astaxanthin can prevent in vitro cell death (apoptosis) in neurons affected in Parkinson’s disease. It was further shown to reduce the production of harmful molecules such as reactive oxygen species (ROS) in MPP+-treated SH-SY5Y cells, thereby decreasing cell damage. Pretreating these cells with astaxanthin (at a concentration of 50 μM) was also observed to protect against oxidative damage. Additionally, astaxanthin helps increase levels of a protective protein called Bcl-2 while decreasing levels of proteins that promote apoptosis, such as α-synuclein and Bax, and reducing the activation of caspase-3, an enzyme that plays a crucial role in apoptosis. At doses of 30 mg/kg, there was an increased number of healthy neurons (tyrosine hydroxylase-positive neurons) and a decrease in damaged neurons (argyrophilic neurons) compared to mice receiving MPTP alone. The evidence found suggests that astaxanthin protects the cells and neurons affected in Parkinson’s disease by regulating proteins that control apoptosis and reducing oxidative stress, being useful in the therapy of neurodegenerative diseases such as Parkinson’s disease [79].

More recently, Lobos et al. (2016) investigated the effects of astaxanthin in primary hippocampal neurons exposed to β-amyloid oligomers, associated with Alzheimer’s disease, (i) reducing the production of mitochondrial reactive oxygen species, which reduces oxidative stress, (ii) inhibiting the activation of NFATc4, which reduces inflammation and cellular damage, and (iii) preventing a decrease in RyR2 gene expression, which maintains the calcium signaling necessary for synaptic function and memory. The results obtained allow us to recognize the protective role against the harmful effects of β-amyloid oligomers by reducing oxidative stress and neuroinflammation. These combined effects suggest that astaxanthin could be a promising strategy to protect the brain from damage associated with Alzheimer’s disease and potentially other neurodegenerative diseases [80].

The effects of astaxanthin on glutamate-induced apoptosis have been investigated in PC12 cells, a cell model of rat medulloblastoma commonly used to study neurodegenerative processes. Excitotoxicity, a pathological process in which neurons suffer damage and death due to overstimulation via excitatory neurotransmitters such as glutamate, was induced in this study. This process occurs when there is excess glutamate in the synaptic cleft, leading to the excessive activation of glutamate receptors on postsynaptic neurons. The consequences of excitotoxicity include neuronal death, which may result from a combination of oxidative damage, mitochondrial dysfunction, and enzyme activation, leading to apoptosis or necrosis. Furthermore, excitotoxicity can cause inflammation and is implicated in various neurodegenerative diseases such as Alzheimer’s and Parkinson’s diseases, amyotrophic lateral sclerosis, and cerebrovascular diseases such as stroke.

The results of the study carried out by Lin et al. (2017) showed that astaxanthin offers protection against glutamate-induced apoptosis by inhibiting intracellular calcium flux and endoplasmic reticulum stress. Although the effects of astaxanthin in in vivo neurodegenerative disease models were not directly investigated in this study, evidence for its effect in PC12 cells was provided. This suggests that astaxanthin could have a potentially relevant neuroprotective mechanism in the context of neurodegenerative diseases, highlighting its potential therapeutic utility [81]. This research corroborates that astaxanthin has the potential to be used as a therapeutic agent in the treatment of neurodegenerative diseases, such as Parkinson’s and Alzheimer’s diseases. It has been shown to be useful as an adjuvant therapy in the prevention and/or delay of neurodegenerative diseases. For example, *Haematococcus pluvialis* and *Chlorella zophingiensis* have shown these effects [82,83]. Finally, astaxanthin can generate anti-Parkinson’s effects in mice, and it has been shown to decrease the activation of microglia in the brain of mice [84,85]. 

In addition to its powerful antioxidant and neurodegenerative properties, astaxanthin also offers protection against damage caused by UV light and age-related diseases, promotes the immune response in various organs, including the liver, kidneys, eyes, and joints, and protects phospholipids from peroxidation. Astaxanthin has also been shown to reduce gastric inflammation and the bacterial load in *Helicobacter pylori* infections, improving cardiovascular risk markers of oxidative stress and inflammation, and having significant potential for the prevention and treatment of several chronic inflammatory disorders, such as cancer, asthma, rheumatoid arthritis, metabolic syndrome, diabetes, and gastrointestinal and liver diseases [76].

Fucoxanthin also presents antioxidant properties. This pigment has aroused interest due to its potential health benefits since, in addition to antioxidant activity, it has anti-inflammatory properties, which are very important in neuroprotection. It also presents immunoprophylactic and antitumor activity, suggesting possible applications in the treatment and prevention of neurodegenerative diseases [32,34,69,86,87]. Fucoxanthin has shown promising preventive and therapeutic potential in various neurological conditions [34]. Hannan et al. (2020) showed a summary of the pharmacological effects, occurrence, effective dose, experimental model, cellular effects, and potential pharmacological mechanism of algal metabolites, in which fucoxanthin constitutes one of the most promising carotenoid pigments [88]. Several studies reported the neuroprotective activity of fucoxanthin [89,90,91,92,93].

Pajot et al. presented the latest studies of functional activities of fucoxanthin (2020–2021), with anti-Alzheimer’s activity being less studied compared to other activities, such as anticancer and antioxidant [32]. Specifically, the study carried out by Yang et al. (2021) of *S. horneri* described fucoxanthin with anti-activity in a β-amyloid oligomers-induced neurotoxicity target [94]. In the study carried out by Lin et al. (2016), it was reported for the first time that fucoxanthin effectively protects against cognitive impairments induced via scopolamine in mice. Furthermore, fucoxanthin significantly reversed the scopolamine-induced increase in acetylcholinesterase (AChE) activity and decreased both choline acetyltransferase activity and brain-derived neurotrophic factor expression. Fucoxanthin was found to directly inhibit AChE with an IC_50_ value of 81.2 μM. Molecular docking analysis suggests that fucoxanthin likely interacts with the peripheral anionic site within AChE, which is consistent with enzyme activity results showing that fucoxanthin inhibits AChE noncompetitively. These authors suggest that fucoxanthin could exhibit great therapeutic efficacy for the treatment of Alzheimer’s disease by acting on multiple targets, including inhibiting AChE and increasing brain-derived neurotrophic factor expression [95]. The effects of fucoxanthin were also investigated in other cellular and animal models of Alzheimer’s disease. The results showed that fucoxanthin penetrates the blood–brain barrier and has effects against neurodegenerative disorders. It can act on multiple targets, including amyloid protein aggregation, oxidative stress, neuroinflammation, neuronal loss, neurotransmission dysregulation, and gut microbiota disorder [94]. These studies provide additional evidence for the potential of fucoxanthin as a neuroprotective agent in neurodegenerative diseases such as head trauma, Parkinson’s disease, and Alzheimer’s disease. Fucoxanthin has shown neuroprotective effects in vitro and has been successfully translated into animal models [88]. This suggests that this compound could be a potential candidate for further evaluation in clinical trials.

Zhang et al. (2017) investigated the neuroprotective effects of fucoxanthin in mouse models of traumatic brain injury. The results of this in vivo study showed that fucoxanthin can provide neuroprotective protection by activating the Nrf2-ARE pathway, reducing oxidative stress and inflammation in the brain after traumatic injury. These findings suggest that fucoxanthin could be a promising therapeutic agent to treat traumatic brain injuries and other neurological conditions associated with oxidative stress and cellular dysfunction [96]. Recently, Chen et al. (2023) provided a comprehensive reference for the application of fucoxanthin in the field of neurology, highlighting its potential as a therapeutic agent for different neurological disorders [34]. It was explained that fucoxanthin exerts its effects by acting on multiple pathways: (1) the regulation of apoptosis and modulation of cell death processes to protect neurons; (2) a reduction in oxidative stress, mitigating the damage caused by reactive oxygen species; (3) the activation of the autophagy pathway, promoting the elimination of damaged cellular components; (4) the inhibition of β-amyloid aggregation, preventing the formation of β-amyloid plaques, which are implicated in Alzheimer’s disease; (5) improving dopamine secretion and increasing neurotransmitter release, which is beneficial in conditions such as Parkinson’s disease; (6) reducing α-synuclein aggregation by reducing the accumulation of proteins associated with neurodegenerative diseases; (7) the attenuation of neuroinflammation after reducing inflammatory responses in the brain; (8) the modulation of the intestinal microbiota due to fucoxanthin’s influence on the gut–brain axis, which affects neurological health; and (9) the activation of a brain-derived neurotrophic factor related to supporting neuronal survival and growth [34]. 

The low bioavailability and limited ability of fucoxanthin to penetrate the blood–brain barrier are drawbacks in the applicability of this pigment for the treatment of neurodegenerative diseases. Future research should focus on developing brain-targeted oral delivery systems. Furthermore, exploring the systemic mechanisms of fucoxanthin metabolism and transport through the gut–brain axis could reveal new therapeutic targets for central nervous system disorders. Dietary supplements incorporating fucoxanthin may offer preventive benefits for neurological conditions [34].

In summary, astaxanthin has shown neuroprotective effects in models of traumatic brain injury, Parkinson’s disease, and hepatic encephalopathy, reducing oxidative stress and neuroinflammation in the brain [96]. Furthermore, fucoxanthin has been shown to protect against neuronal damage in models of Alzheimer’s disease, traumatic brain injury, cerebral ischemia, and hepatic encephalopathy, improving neurological function and reducing oxidative stress and brain inflammation [68]. These findings suggest that astaxanthin and fucoxanthin have significant therapeutic potential in the development of drugs for the treatment of neurodegenerative diseases.

These results suggest a promising therapeutic potential for both astaxanthin and fucoxanthin in the development of dietary supplements of drugs for neurodegenerative diseases. Furthermore, marine microalgae and macroalgae are natural sources of these bioactive compounds, which opens industrial possibilities for their large-scale extraction. The marine biotechnology industry is investigating efficient methods of extraction and production of these compounds, which could lead to their use in pharmaceutical products and nutritional supplements aimed at the treatment and prevention of neurodegenerative diseases.

As mentioned, evidence suggests that both fucoxanthin and astaxanthin could offer significant neuroprotective benefits [64,68]. However, human clinical trials of fucoxanthin and astaxanthin are still limited, and more research is needed, especially in the field of neurodegenerative diseases. In fact, when searching for this topic for astaxanthin, little information has been found [97] [https://clinicaltrials.gov/search?cond=astaxanthin (accessed on 10 July 2024)] even though thirty-two references appeared, compared to five references for fucoxanthin [97] [https://clinicaltrials.gov/search?cond=Fucoxanthin (accessed on 10 July 2024)].

Related to astaxanthin, studies on its formulation to improve its bioavailability and pharmacokinetics have been performed. Related to the main topic of this overview, astaxanthin has been studied, and its action on inflammation and cognition, and as an antioxidant, being especially useful for patients suffering from Alzheimer’s disease, has been claimed. Regarding age-related diseases, this pigment has been studied in skin aging, skin health, and cardiovascular diseases (i.e., stroke), as it is considered an excellent supplement for athletes.

For both astaxanthin and fucoxanthin, some information focused on metabolic syndrome, insulin sensitivity, cognitive function, and body weight control has been found. Directly focused on fucoxanthin, the role of dietary-rich supplements for liver health conditions has also been studied. Related to the main topic of this review, the effects of this pigment on cognitive function in healthy older subjects have been studied and claimed. The role in cognitive function has also been associated with the gaming performance of video-gamers. 

The lack of clinical trials may change in the near future due to the interest in both pigments. Table 3 summarizes the most relevant clinical trials, including those within the field of brain-related diseases.

### 3.2. Use of Algae-Derived Pigments in Nutraceuticals

Several authors reported in vitro and in vivo studies supporting the development of new dietary supplements or carotenoid-based drugs [68,98]. Inflammatory stimuli appear to be related to a dysfunction of the mitochondrial metabolism, which can induce chronic inflammation. Neuroinflammation leads to neurodegenerative pathologies [99]. The xanthophyll family, which includes astaxanthin and fucoxanthin, can act on reactive oxygen molecules to normalize mitochondrial functions, reducing chronic inflammation. Furthermore, recent studies have focused on the relationship between the intestinal flora and the central nervous system, triggering a bidirectional communication called the “gut–brain axis”. These findings are promoting new research trends involving neurodegenerative disorders and gut microbes. Carotenoids, polysaccharides, and terpenes from natural marine products showed regulators of the anti-inflammatory effect and apoptotic regulation. These effects contribute to presenting the aforementioned natural compounds as a promising source of functional foods targeting the microbiota that have a modulating role of deregulated brain–gut mediators, improving neuroprotective activity [100]. Finally, recent research has explored the development of nanoformulations and nano-drug delivery systems based on marine carotenoids to optimize their oxidative stress activity and bioavailability [101,102].

Looking at the data up to 2023 reveals that the fucoxanthin market has mainly expanded in the Asia-Pacific region, followed by the United States and Europe. The value of this market was 209.45 USD with projections of 294.72 Mn USD by 2030. The main application of fucoxanthin is in the food industry, but there are also encouraging figures in cosmetics and pharmaceuticals. Although the priority market is for food pigments of natural origin, data on biological activity are increasing the demand for the supplements sector, and in particular, there is a growing demand for the treatment of obesity and depression. In the cosmetics sector, fucoxanthin is marketed for skin care and hair care [103].

The astaxanthin market shows similar projections to that of fucoxanthin, with the same market dominance in the Asia-Pacific region, followed by the United States and Europe. However, the market is significantly larger, with sales of 769.56 million USD in 2022 and an expectation of 1365.75 million USD in 2029. In addition, it is a more diversified market, with sales structured in the three traditional sectors (food, pharmaceuticals, and cosmetics) and also food supplements and aquaculture. Market expansion is expected in the cosmetic sector [104].

Despite the encouraging market data, it must be remembered that regulatory aspects and other production cost difficulties remain the predominant challenges for companies that market the aforementioned products. There are technical difficulties related to the separation and purification aspects that require further investigation but will improve compliance with REACH regulations in Europe and the FDA in the USA [105].

## 4. Future Perspectives

Although, in recent decades, a significant number of marine compounds with promising neuroprotective properties have been identified, few of them have reached clinical trials. Further research, including additional in vivo studies and human clinical trials, is needed to confirm these effects and determine the efficacy and safety of their use as food supplements or even drugs. When comparing the neuroprotective potential of fucoxanthin and astaxanthin, it can be observed that each of these marine pigments has unique properties, but in terms of evidence and studies, fucoxanthin seems to be the most promising compound. This pigment has been extensively studied and has been shown to be effective in protecting against the main key processes in neurodegeneration. It seems that its neuroprotection is due to several mechanisms. Among them, its ability to reduce oxidative stress stands out, which is an important factor in neuronal cell death and neurodegenerative diseases’ progression. In addition, it can inhibit neuroinflammation, another critical factor in the progression of these diseases. It has also been observed to promote cell survival, neurogenesis, and synaptic plasticity, which contributes to the repair and maintenance of neuronal tissue. Preclinical studies have shown that fucoxanthin protects neurons against various types of neurotoxic stress, including oxygen and glucose deprivation, hydrogen peroxide, glutamate, and beta-amyloid, which are relevant in the context of neurodegenerative diseases, and brain injuries [106]. However, despite the promising results in both in vitro and in vivo studies, clinical research on fucoxanthin is still limited. Only one significant clinical study has been conducted in recent years, focused on obesity [106]. Further studies are needed to address the efficacy and safety in humans of this marine compound. Astaxanthin is one of the most valuable microalgae products, and its future seems also to be very promising. However, it has not yet been explored in the food industry due to a lack of adequate data in terms of long-term consumption [65]. Furthermore, the cost of naturally occurring astaxanthin is high, so it is necessary to look for new ways to reduce this cost through the design of new technological processes [65]. In general, it has been observed that work must be done to find efficient methodologies to reach good amounts of both pigments in order to make their production efficient enough to be rentable.

## 5. Conclusions

The identification of new therapeutic targets and treatment approaches remains an active area of research in the field of neurodegenerative diseases. Fucoxanthin and astaxanthin have been studied for their role as potential nutraceuticals and drugs with activities against neurodegenerative diseases. Of the two pigments analyzed, fucoxanthin has been widely studied for its bioactive activities, including its neuroprotective properties. This pigment has been shown to be effective in protecting against oxidative stress and inflammation, two key processes in neurodegeneration. Its potential is supported by solid scientific evidence, but it requires more clinical studies to confirm its effectiveness and safety for human therapeutic applications. The methods of cultivation and extraction of fucoxanthin and astaxanthin are essential to take advantage of the neuroprotective benefits of these compounds. Advanced cultivation techniques in photobioreactors, together with environmentally friendly extraction methods, guarantee the production of high-quality, safe, and effective products for therapeutic applications.

## Figures and Tables

**Table 1:** Chemical structures of the most relevant biopigments present in marine algae.

Pigments	Structure	Chemical Class
Fucoxanthin	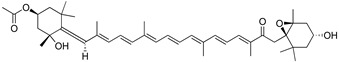	Carotenoids/xanthophylls
Astaxanthin	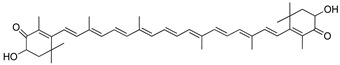	Carotenoids/xanthophylls
Lutein	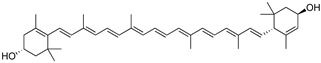	Carotenoids
Canthaxanthin	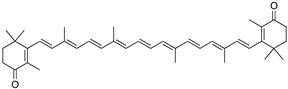	Carotenoids
Zeaxanthin	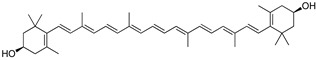	Carotenoids
β-Cryptoxanthin	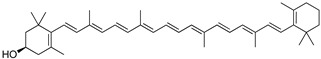	Carotenoids
Chlorophyll A/B	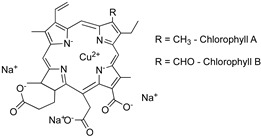	Chlorophyll
Phycoerythrin	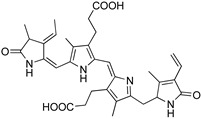	Phycobiliproteins
Phycocyanin	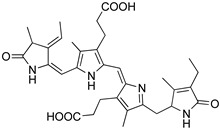	Phycobiliproteins

**Table 2:** Most relevant species of algae that produce astaxanthin and fucoxanthin.

Pigment	Species	Content (mg g^−1^ DW)	References
Astaxanthin	*Haematococcus pluvialis*	27–50	[28,35]
	*Neochloris wimmeri*	6.000	[28]
	*Chlorococcum*	2.000	
	*Chlorella zofingiensis*	0.010	
Fucoxanthin	*Phaeodactylum tricornutum*	15.71	[36]
	*Odontella aurita*	14–15	
	*Chaetoceros calcitrans*	5.130	
	*Isochrysis galbana* T-ISO	3.180	
	*Nitzchia laevis*	2.370	
	*Isochrysis galbana*	2.190	
	*Chaetoceros calcitrans*	2.330	
	*Odontella sinensis*	1.180	
	*Undaria pinnatifida*	0.876–1.277	
	*Skeletonema costatum*	0.360	
	*Eisenia bicyclis*	0.109	
	*Kjellmaniella crassifolia*	0.152	
	*Sargassum horneri*	0.020	
	*Saccharina japonica*	0.030	
	*Sargassum fusiforme*	0.010	
	*Cystoseira hakodatensis*	0.0041	
	*Phaeodactylum tricornutum*	0.07	

**Table 3:** Most relevant clinical trials for both astaxanthin and fucoxanthin.

Pigment	Title	Conditions	Year	Locations
Astaxanthin	Astaxanthin formulation bioavailability	Bioavailability	2015	USF Health: College of Medicine, United States
	Effect of astaxanthin on patients with Alzheimer’s disease	Formulations to evaluate the possible benefit on Alzheimer’s disease	2018	Kaohsiung Medical University Chung-Ho Memorial Hospital, Taiwan
	Bioavailability of astaxanthin formulations	Healthy volunteers	2018	Bert W. Strassburger Lipid Center, Sheba Medical Center, Israel
	Astaxanthin, exercise inflammation, and skin health	Inflammatory response Metabolic disturbance Immune suppression	2022	Appalachian State University Human Performance Lab, North Carolina Research Campus, United States
	Astaxanthin reduces exercising heart rate	Metabolic flexibility	2021	University of North Alabama, Department of Kinesiology, United States
	Astaxanthin supplementation in cyclists	Athletic performance	2010	Maastricht University, Netherlands
	Safety and pharmacokinetics of phaffia rhodozyma astaxanthin	Absorption; chemicals	2018	Sourasky Medical Center, Israel
	Impact of astaxanthin on cognition in recreationally active females	Mental fatigue	2024	University of North Alabama, United States
	Effects of isoflavone combined with astaxanthin on skin aging	Aging Photo-aging	2015	Seoul National University Hospital, Republic of Korea
	Astaxanthin for management of inflammation in knee osteoarthritis	Osteoarthritis, knee Joint inflammation	2022	Prisma Health, Columbia, United States
	Astaxanthin effects on osteoarthritis-associated pain and inflammatory indicators	Osteoarthritis, knee	2021	Saint Luke’s Hospital of Kansas City, United States
	Efficacy and safety of astaxanthin for volunteers with refraction errors	Refractive errors	2021	Not provided
	Oral supplementation of astaxanthin on skin photo-aging, hydration, and elasticity	Photo-aging	2023	Integrative Skin Science and Research, Sacramento, United States
	Effect of astaxanthin in moderate to severe knee osteoarthritis	Osteoarthritis, knee	2022	Bangabandhu Sheikh Mujib Medical University, Bangladesh
	Clinical trial of anti-oxidant astaxanthin in insulin-resistant subjects	Metabolic syndrome X	2016	Altman Clinical and Translational Research Institute (ACTRI), United States
	Physiological and molecular influences of astaxanthin supplementation on heat strain in humans	Body temperature regulation	2014	Sheba Medical Center, Israel
	Clinical trial of astaxanthin formulation with exercise in elderly people with sarcopenia	Sarcopenia	2015	Fred Hutchinson Cancer Research Center Prevention Center, United States University of Washington Medical Center, United States
	A study to evaluate the effect of astaxanthin in healthy participants	Healthy	2022	SGS Stephens, Inc, Richardson, United States
	Effect of astaxanthin supplementation on plasma malondialdehyde levels and NIHSS of stroke patients	Cerebral stroke Malondialdehyde Oxidative stress	2010	Department of Nutrition University of Indonesia, Indonesia
	Evaluating astaxanthin bioavailability and a new technology for improving it, using natural food materials only	Bioavailability	2018	Rambam Health Campus, Israel
	Lipid-lowering effects of an astaxanthin supplement in volunteers with mild dyslipidaemia	Dyslipidemias	2014	Centre Nutrition Clinique Naturalpha, France
	The effect of astaxanthin on oxidative stress indices in patients with polycystic ovary syndrome	PCOS	2020	Shariati Hospital, Islamic Republic of Iran
	Study of the efficacy and safety of antioxidant astaxanthin as an adjuvant therapy for community-acquired pneumonia patients	Community-acquired pneumonia	2024	Elmatarya Teaching Hospital, Egypt
	Effect of CEAG on inflammation and endothelial function	Inflammation Endothelial dysfunction	2018	Lundquist Institute for Biomedical Innovation at Harbor UCLA Medical Center, United States
	The benefits of astaxanthin as an add-on therapy in the management of painful diabetic neuropathy patients	Painful diabetic neuropathy	2020	Bethesda Hospital Yogyakarta, Indonesia
	Oral astaxanthin and semen quality, fertilization, and embryo development in assisted reproduction technique procedures	Infertility, male	2014	Division of Ob/Gyn, University Medical Centre Ljubljana, Slovenia
	Astaxanthin (2 mg) + lycopene (1.8 mg) + d-*alpha*-tocopherol (10 iu) for the treatment of skin aging	Skin aging Wrinkles	2018	PDC Building, Philippines
	Positive effects of Haematococcus astaxanthin on oxidative stress and lipid profiles in overweight and obese adults	Healthy Overweight Obesity	2010	Clinical Research Institute Seoul National University Hospital, Republic of Korea
	Effect of omega-3 fatty acid supplementation on dry-AMD progression	Age-related macular degeneration	2022	Shanghai General Hospital, Shanghai Jiao Tong University, China
	Use of ritmonutra in subjects affected by supraventricular ectopic beats without structural heart disease	Arrhythmia	2013	Policlinico San Pietro, Italy Policlinico San Donato, Italy
	Combined effects of bioactive compounds in lipid profile	Hyperlipidemia Low-density- Lipoprotein-type Elevated triglycerides	2012	Hosp. Universitario San Joan, Spain
	Nutritional supplement’s effects on cognition	Dietary supplement Cognition Healthy	2023	Clinical Research Australia, Australia
Fucoxanthin	Effect of fucoxanthin on metabolic syndrome, insulin sensitivity, and insulin secretion	Metabolic syndrome	2019	Instituto de Terapéutica Experimental y Clínica, Mexico
	Effect of brainphyt, a microalgae-based ingredient on cognitive function in healthy older subjects	Healthy aging Dietary supplement Cognitive impairment Neuroprotection Mood Stress Sleep	2021	Atlantia Clinical Food trial, Ireland
	Microalgae extract phaeosol combined with exercise for healthy overweight women: efficacy on body weight management	Overweight and obesity Body-weight changes Healthy lifestyle Exercise Dietary supplement	2021	Exercise & Sport Nutrition Lab, United States
	Oral dietary fucoxanthin-rich supplement for liver health	Non-alcoholic fatty liver	2018	
	Efficacy of a microalgae extract, phaeosol, combined with a natural stimulant on the cognitive function and gaming performance of video-gamers	Cognitive function Video-gamers	2021	Exercise & Sport Nutrition Lab, United States

## Data Availability

Not applicable.
